# Application potential of bone marrow mesenchymal stem cell (BMSCs) based tissue-engineering for spinal cord defect repair in rat fetuses with spina bifida aperta

**DOI:** 10.1007/s10856-016-5684-7

**Published:** 2016-02-19

**Authors:** Xiaoshuai Li, Zhengwei Yuan, Xiaowei Wei, Hui Li, Guifeng Zhao, Jiaoning Miao, Di Wu, Bo Liu, Songying Cao, Dong An, Wei Ma, Henan Zhang, Weilin Wang, Qiushi Wang, Hui Gu

**Affiliations:** Key Laboratory of Health Ministry for Congenital Malformation, Shengjing Hospital, China Medical University, No.36, Sanhao Street, Heping District, Shenyang, 110004 China; Department of Pediatric Surgery, Shengjing Hospital, China Medical University, Shenyang, China; Department of Blood Transfusion, Shengjing Hospital, China Medical University, Shenyang, China

## Abstract

Spina bifida aperta are complex congenital malformations resulting from failure of fusion in the spinal neural tube during embryogenesis. Despite surgical repair of the defect, most patients who survive with spina bifida aperta have a multiple system handicap due to neuron deficiency of the defective spinal cord. Tissue engineering has emerged as a novel treatment for replacement of lost tissue. This study evaluated the prenatal surgical approach of transplanting a chitosan–gelatin scaffold seeded with bone marrow mesenchymal stem cells (BMSCs) in the healing the defective spinal cord of rat fetuses with retinoic acid induced spina bifida aperta. Scaffold characterisation revealed the porous structure, organic and amorphous content. This biomaterial promoted the adhesion, spreading and in vitro viability of the BMSCs. After transplantation of the scaffold combined with BMSCs, the defective region of spinal cord in rat fetuses with spina bifida aperta at E20 decreased obviously under stereomicroscopy, and the skin defect almost closed in many fetuses. The transplanted BMSCs in chitosan–gelatin scaffold survived, grew and expressed markers of neural stem cells and neurons in the defective spinal cord. In addition, the biomaterial presented high biocompatibility and slow biodegradation in vivo. In conclusion, prenatal transplantation of the scaffold combined with BMSCs could treat spinal cord defect in fetuses with spina bifida aperta by the regeneration of neurons and repairmen of defective region.

## Introduction

Neural tube defects (NTDs) are common congenital malformation. Spina bifida and anencephaly are the most common and severe forms of NTD affecting about 1 in 2000 live births worldwide [1]. In China, approximately 80,000–100,000 new-borns each year are diagnosed to have NTDs [2]. So far, Neural tube defects treatment has no breakthrough progress, in addition, Hydrocephalus, lower limb dysfunction and urinary incontinence is still serious postoperative complications [3]. So it is important to give appropriate treatment at early stage of gestational period to minimize continuous damages of the exposed spinal cord.

Fetal cellular therapy is a treatment option for a variety of birth defects, and it has been employed in the treatment of congenital haematologic disorders and immunodeficiency disease [4–6], and also in experimental foetal lamb model of myelomeningocele (MMC) [7]. We have previously shown that the deficiency of sensory and motor neuron is a primary anomaly in spinal cord of spina bifida aperta, which suggests that neuron replacement therapy based on neuron regeneration or cell replacement of the defective spinal cord is a promising approach to achieve a better functional outcome in spina bifida [8, 9]. Bone marrow-derived mesenchymal stem cells (BMSCs) have the capacity to self-renew, easy isolation, implanted less adverse reaction and multipotent differentiation potential such as skeletal muscle, lung, vascular, neuron, astrocyte, bone, intestinal and liver cells in response to different factors [10, 11]. A large number of studies have shown that BMSCs can trans-differentiated into neurons in vitro and in vivo [12–16]. In recent studies, BMSCs have also shown healing capability by preventing fibrosis and improving angiogenesis, which could have a role in tissue repair and tissue regeneration [17]. Therefore, we successfully established a new approach to inject BMSCs into defective spinal cord for the potential treatment of spina bifida aperta. Our data indicated that BMSCs survived, migrated, and differentiated into neurons in the spinal cord [18]. In addition, injected BMSCs expressed and induced the surrounding spinal tissue to express neurotrophic factors [19]. Our results suggest that prenatal BMSCs transplantation can be used to treat spinal neuron deficiency in NTDs and might serve as a potential treatment option for other congenital anomalies. However, due to the limited injection space, the amount of surviving BMSCs was not enough to reconstruct the defective spinal cord. In this context, tissue engineering has emerged as an effective alternative therapy to repair the spinal cord defects. Tissue engineering is an interdisciplinary field involving cell biology and materials science that works toward the development of biological substitutes to restore, maintain or improve tissue function [20, 21]. The basic principle of tissue engineering involves seeding cells onto a biodegradable scaffold to generate new tissue [22–24]. The choice of the biomaterial is critical for the success of such an approach in spinal cord repair. The ideal scaffold should possess suitable mechanical properties, biocompatibility, biodegradability, three-dimensional structure, and promote cell growth. A biocompatible material does not cause toxic or injurious effects on biological systems [25]. Because of its acceptable biodegradability [26], high biocompatibility [27], chemical similarity to the structure of extracellular matrix (ECM), anti-microbial activity, [28] and capacity to produce porous scaffolds, [29] chitosan has been widely studied as a biomaterial for tissue-engineering applications [30–32]. Its association with gelatin, a source of collagen type I, enhances the mechanical and cell adhesion in culture, due to the affinity of the cells for the adhesion proteins [33–35]. Chitosan has also other useful biological properties such as hemostasis and acesodyne activity, wound healing property, permeability to oxygen, reducing scars, antifungal and bacteriostasis character which make it important as a dermal substitute, and has similar structure to glycosaminoglycans (GAGs) to help wound healing [36, 37].The chitosan/gelatin/PAG scaffold as a suitable material having proper conductivity, mechanical properties and biocompatibility that may be appropriate for different biomedical applications such as scaffold material in tissue engineering for neural repair [38]. Tissue engineering materials could potentially lead to new therapeutic strategies for nervous tissue injuries as well as provide novel investigative tools for biological research [39]. In a word, tissue engineering are changing the way we think about how we might treat patients born with serious congenital malformations [40].

Therefore, in present study, we proposed a strategy for tissue engineering, by the implantation of a chitosan–gelatin scaffold seeded with labeled eGFP-BMSCs in fetal rats with spina bifida by the combined techniques of microsurgery and fetal surgery.

## Materials and methods

### Experiment animals

Outbred Wister rats of 10–12 weeks of age (250–300 gm) were purchased from the animal centre of China Medical University. The appearance of vaginal plugs in the female rat the morning after mating was timed as the embryonic day 0 (E0). Spina bifida aperta were induced with a single intragastric retinoic acid (140 mg/kg body weight; Sigma) administration on E10 as previously described [9, 18, 19, 41]. All the animal experiments were performed with the approval obtained from the ethics committee of China Medical University.

### Isolation, culture expansion and transfection of BMSCs

BMSCs were isolated from 4-week-old Wister rat following previously published protocol [14, 19]. Rat BMSCs were cultured in DMEM/F12 (Gibco) supplemented with 10 % fetal bovine serum (FBS; Hyclone) and 100 IU/ml penicillin to 100 μg/ml streptomycin (Gibco) on 25 cm^2^ tissue culture flasks (BD Biosciences). Primarily isolated BMSCs were defined as P0, at confluency, cells were passaged (1 in 2 dilution) with fresh medium. Cultured BMSCs expressed CD90, CD44, CD73 and CD29 but not CD34 and CD45 as revealed by flow cytometry using specific antibodies (551401, 550974, 551123, 562154, 560932, 554878; BD Biosciences) following previously published methods. After primary antibodies incubation, sections were washed three times with PBS followed by incubation with Alexa Fluor 488-conjugated goat anti-rabbit IgG antibody (Invitrogen) and TRITC-conjugatedgoat anti-mouse IgG (AP124R; Millipore) in 10 % FBS-PBST for 1 h at room temperature. After washing, sections were stained with DAPI (C1002; Beyotime Institute of Biotechnology), then mounted with anti-fade mounting medium (P0126; Beyotime Institute of Biotechnology). The expression of CD90 was used to estimate the purity of BMSCs [36, 42]. Twenty-four hours before transplantation, BMSCs were transfected with eGFP expression adeno-5 vector (SinoGenoMax.Co., Ltd, Beijing, China) (100 pfu/1 cell), for the visualization of BMSCs after transplantation into rat fetus.

### Preparation and characterization of the chitosan–gelatin scaffolds and cell-scaffold construct

#### Preparation of chitosan–gelatin scaffolds

**Fig. 1:**
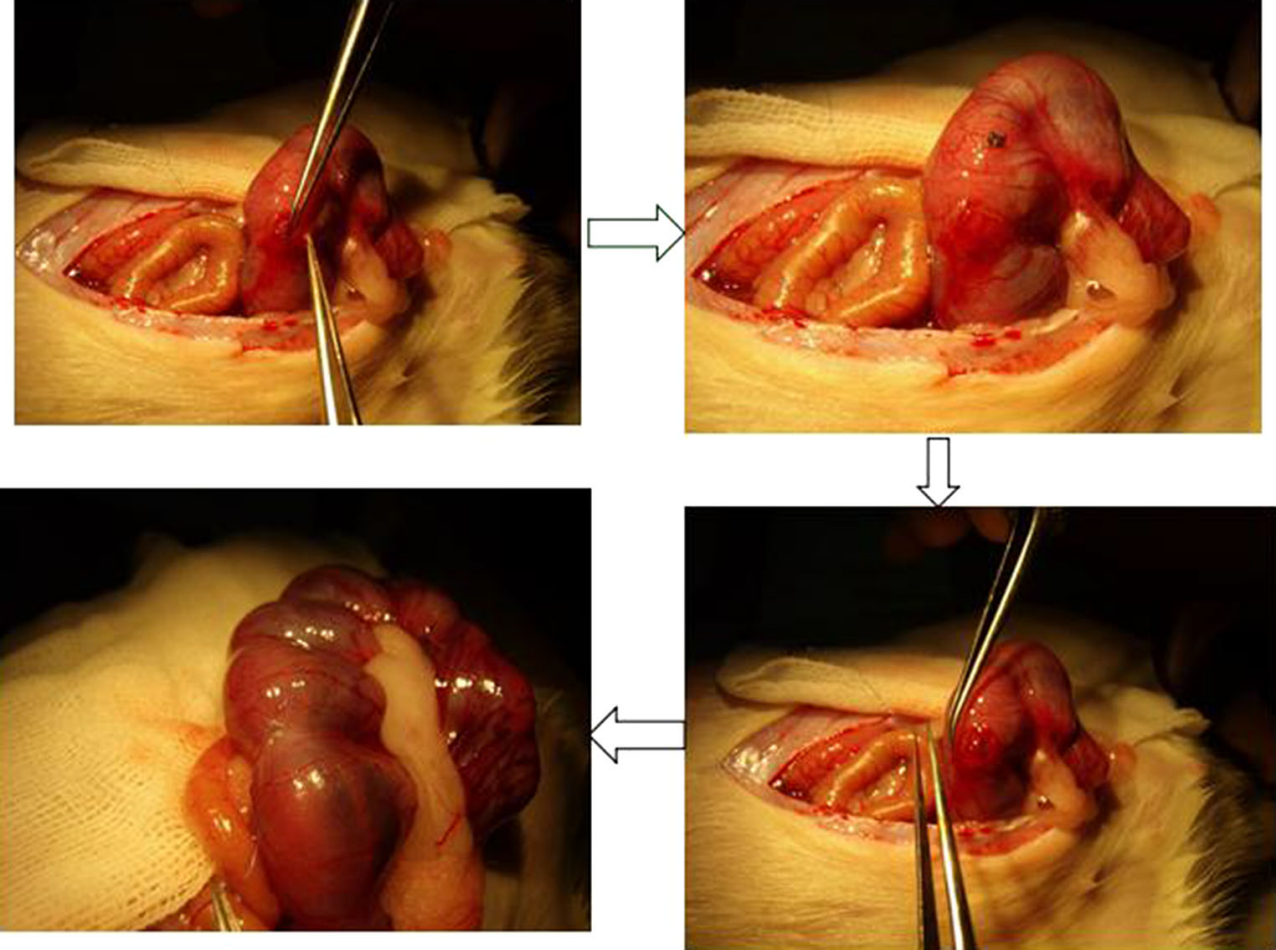
Operation process of fetal surgery and implantation. The *black arrow* indicate the cell-scaffold construct

Chitosan–gelatin scaffolds were obtained via a freeze-drying technique and genipin (Sigma) cross-linking as previously described [43]. In this method, the solvent was removed by sublimation, and the corresponding empty spaces became the pores of the scaffold. The scaffolds were produced from natural polymers, chitosan (Sigma) with a degree of de-acetylation of 85 % and type A porcine skin gelatin (Vetec,RJ, Brazil). Chitosan 0.7 % (w/v) solution and gelatin 0.7 % (w/v)solution were dissolved in 0.1 M acetic acid (Vetec, RJ, Brazil) separately. These two distinct solutions rested for 24 h at room temperature, and they were then mixed at a 3:1 (chitosan/gelatin) ratio. Subsequently, the chitosan–gelatin blend was cross-linked with 25 % glutaraldehyde solution at a 0.1 % concentration. The final solution was under agitation for 1 h. One millilitre of the homogenous mixture of chitosan, gelatin and glutaraldehyde was poured into each well of cell culture plate (24-well), used as a template to mould cylindrical standard discs of chitosan–gelatin sponges. After overnight air drying, the plates were incubated at 37 °C for 24 h. The plates were then frozen at −20 °C for an additional 24 h, and the scaffolds were lyophilised. Next, the lyophilized scaffolds were resuspended in a 25 % glutaraldehyde solution at a 0.1 % concentration (1 mL/well), and the plates were shaken for 30 min. The procedure (heat, freezing and lyophilisation) was then repeated. Finally, 1 mL/well of 100 % alcohol was added, and the samples were left to completely dry. Eight-millimetre diameter discs, 2.5-mm thick, with a spongy texture, were cut into 4 parts. The standard-size scaffolds, slices of 4 mm in radius (Fig. 1a), were used in vitro and in vivo procedures. The samples were placed at the bottom of a 24-well cell culture plates, sealed and sterilized at Nuclear Development Technology Centre/Nuclear Energy National Commission (CDTN/CNEN) by γirradiation, ^60^Co, 20 Grays.

#### Characterization of swelling degree and surface density

The scaffold was dried to constant weight, the length and width was measured by micrometer and the thickness was measured by spiral micrometer and finally calculate the volume as V_dry_, the weight was measured by photoelectric analytical balance as W_dry_, and then be placing in the PBS buffer solution for 48 h to fully swelling, using filter paper to absorb surface moisture and rapidly weighing as Wwet, the formula of swelling rate and apparent density calculation is as follows:

1 $${\text{SW}}_{\text{swelling rate}} = \, {{{\text{W}}_{\text{wet}} {-}{\text{W}}_{\text{dry}} } \mathord{\left/ {\vphantom {{{\text{W}}_{\text{wet}} {-}{\text{W}}_{\text{dry}} } {{\text{W}}_{\text{dry}} \times 100\;\% }}} \right. \kern-0pt} {{\text{W}}_{\text{dry}} \times 100\;\% }}$$

2 $$\rho_{\text{apparent - density}} = {\text{W}}_{\text{dry}} /{\text{V}}_{\text{dry}} \times 100\;\%$$

3 $${\text{V}}_{\text{dry}} = {\text{D}}_{{{\text{dry}}\_{\text{thickness}}}} \times {\text{L}}_{{{\text{dry}}\_{\text{length}}}} \times {\text{W}}_{{{\text{dry}}\_{\text{width}}}}$$

#### Measurement of porosity

Four scaffolds were randomly selected, soaking in anhydrous alcohol, until they were filled out and using filter paper to absorb the alcohol on the surface and rapid weighing, measuring three times to get the average value is denoted as Q. The density of anhydrous ethanol denoted as P_eth_, the calculation formula of porosity is as follows:

4 $${\text{P}}_{\text{d}} = ({\text{Q}} - {\text{W}}_{\text{dry}} )/\uprho_{\text{eth}} \cdot {\text{V}}_{\text{dry}} \times 100\;\%$$

#### Measurement of aperture

The morphological characterization of scaffolds was performed by scanning electronic microscopic (SEM) analysis. The samples of chitosan–gelatin scaffolds and cell-scaffold constructs were fixed in 2.5 % glutaraldehyde (0.1 mol/L phosphate buffer—PBS, pH 7.4)for 48 h and post fixed with 1 % osmium tetroxide for 2 h. After dehydration, the critical-point dried in liquid CO_2_ (CPD-020 Balzers) was performed. The samples were mounted on metallic holders, sputter-coated with gold and observed in a Zeiss DSM 950 scanning electron microscope, at an accelerating voltage of 15 kV and 750 mA. To observe the surface morphology of chitosan–gelatin scaffolds on the console and image acquisition, selecting 20 apertures randomly and calculates the size, and calculate the average value.

#### Seeding and culture of BMSCs in chitosan–gelatin scaffold

Before seeding, cells were trypsinized, centrifuged, resuspended in small aliquot of fresh medium, GFP positive cell numbers were counted. By the method of microinjection, the cells are more evenly distributed inside the scaffold. And then they were plated in 24-well culture plates (Nunc) in DMEM/F12 (Gibco) supplemented with 10 % fetal bovine serum. Before transplantation, they were incubated for 48 h at 37 °C with 5 % CO_2_.

#### Proliferation assay

To evaluate the viability of cells in the scaffold. The scaffold seeded with BMSCs was cultured in DMEM/F12 (Gibco) supplemented with 10 % fetal bovine serum (FBS; Hyclone) and 100 IU/ml penicillin to 100 μg/ml streptomycin (Gibco), and medium changes everyday. After 1 and 2 week, the scaffold was stained by fluorescein diacetate (FDA) (BioBas)- propidium iodide (Pl) (Sigma). The scaffold was removed from the culture, and it was washed with PBS 10 min twice. The scaffold was stained with FDA (1ug/ml), it was incubated for 15 min at 37 °C. After washing with PBS 10 min twice, the scaffold was stained with PI (1 mg/ml) for 1 h at room temperature. After washing with PBS 10 min twice, image was taken with fluorescence microscope connected to a CCD camera (Nikon).

### Foetal surgery and implantation

We developed a new approach to transplant the cell-scaffold construct into fetuses with spina bifida aperta using combined techniques of fetal surgery and microsurgery. Retinoic acid treated pregnant rats of E16 were anaesthetized with pentobarbitone sodium (40 mg/kg body weight). An incision was made in the abdominal wall, and the uterine horn was exteriorized. To relieve uterine spasm, the uterus was covered with wet gauze immersed with warm physiologic saline and atropine (0.1 mg/kg body weight) was given intraperitoneally. Under the microscope the position of defective spinal dord of fetus was identified through the wall of uterus. Then 7–0-nylon purse-string suture and a small incision were made on the wall of uterus. The amniotic sac was opened and the defective region of spinal cord was exposed. The cell-scaffold construct was implanted into the defective region of spinal cord under the microscope. After implantation, the fetuses were returned to the uterus, and the wound of the uterus was closed (Fig. 1). In average, 2–3 fetuses could be implanted without compromising the survival of the fetuses. The pregnant rats recovered from the anesthesia within 1 h and were returned to their home cage.

### Sample collection

The pregnant rats on E20 were reanesthetized with an overdose of pento-barbitone sodium, and the fetuses that had been transplanted were harvested by caesarean section. Fetuses were perfused transcardially with 15 ml physiologic saline followed by 25 ml 4 % paraformaldehyde. The lumbosacral spinal column containing muscle, spinal cord and subcuta-neous tissue was dissected and post-fixed in the same fixative for 24 h at 4 °C, followed by cryopreservation in 20 % sucrose for 24 h. The chitosan–gelatin scaffolds seeded with labeled eGFP-BMSCs in the spinal column were observed under stereomicroscope (Leica). The spinal column were then sectioned into 30 μm serial transverse sections, and all GFP positive BMSCs in the spinal column were observed and counted under fluorescence microscope (Nikon, Tokyo, Japan). All the sections were also stained with DAPI, and only those GFP positive cells that contained a nucleolus were counted to avoid the same GFP cell being counted more than once. Image was taken with C1 confocal microscope (Nikon). Sections with GFP positive BMSCs observed were marked and kept at −80 °C in the dark for further immuno-fluorescence staining.

### Immunofluorescence

To evaluate the differentiation potentials of transplanted BMSCs in the spinal column, sections were analysed by immunofluorescence using antibodies against molecular markers of neural stem cell and neuron. Primary antibodies used were mouse anti-nestin (1:100) (MAB353;Millipore) and mouse anti-Tubulin (1:200) (MAB3402; Millipore). After primary antibodies incubation, sections were washed three times with PBS followed by incubation with Alexa Fluor 488-conjugated goat anti-rabbit IgG antibody (Invitrogen) and TRITC-conjugated goat anti-mouse IgG (AP124R; Millipore) in 10 % FBS-PBST for 1 h at room temperature. After washing, sections were stained with DAPI (C1002; Beyotime Institute of Biotechnology), then mounted with anti-fade mounting medium (P0126; Beyotime Institute of Biotechnology).

## Results

### BMSCs culture characters and eGFP transfection

**Fig. 2:**
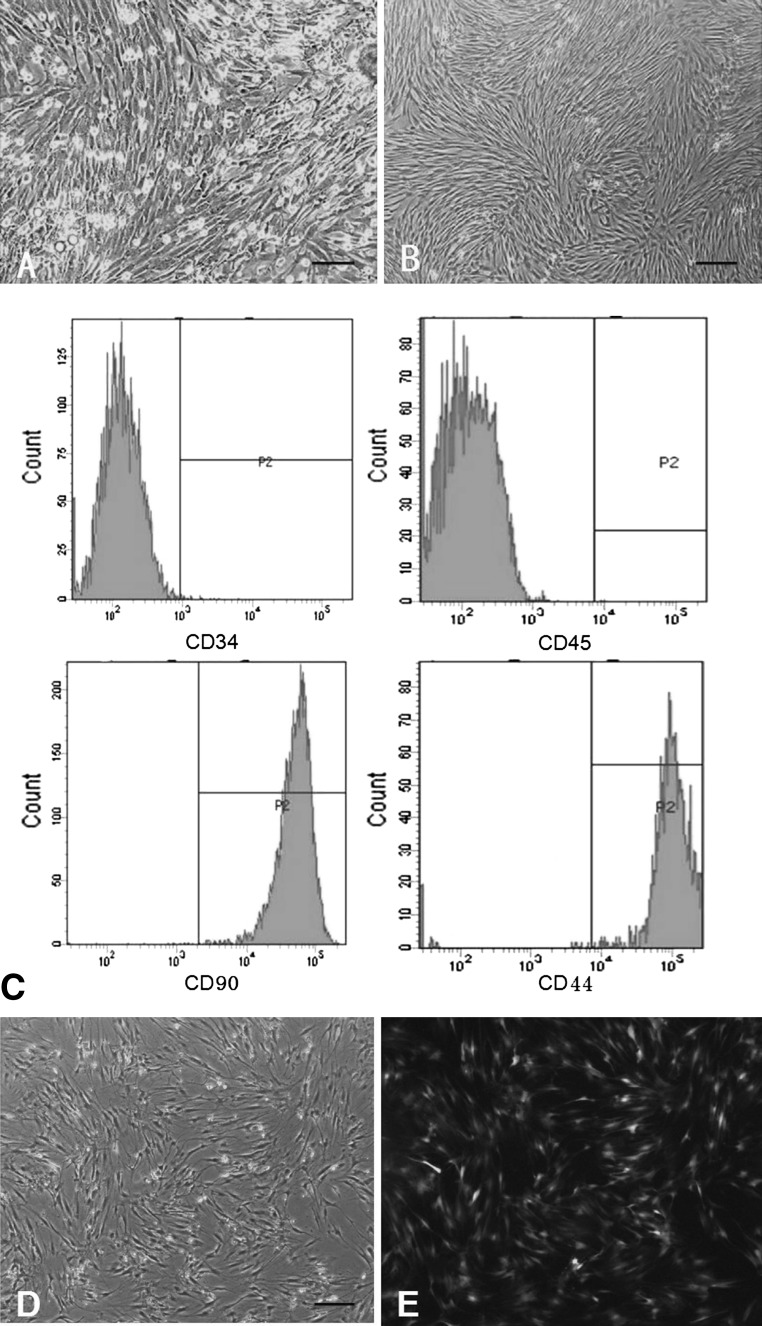
**a** The LM micrograph of BMSCs after adherent 24 h; **b** the LM micrograph of BMSCs to P3 generation showing the characteristic of plastic adherence and spindle shape. **c** The result of flow cytometry histograms demonstrating high expression of non-hematopoietic markers of CD90 and CD44 and low expression for CD34 and CD45. **d**,**e** The figure **d**, **e** was in the same perspective, after GFP adenovirus transfection, the efficiency rate can reach above 90 %. The scale of all the pictures are 100 μm

BMSCs presented typical spindle-shape morphology with adherence to plastic and organisation in monolayer (Fig. 2). The phenotypic characterisation of the BMSCs indicated high expression of non-hematopoietic markers, CD90 (95 %) and CD44 (94 %). Additionally, no CD34 and CD45 expression was present in 97 and 98 % of the cells (Fig. 2c). Thus, the isolated cells met the criteria used to define BMSCs.

### Characterization of the physicochemical properties of chitosan gelatin scaffolds

**Fig. 3:**
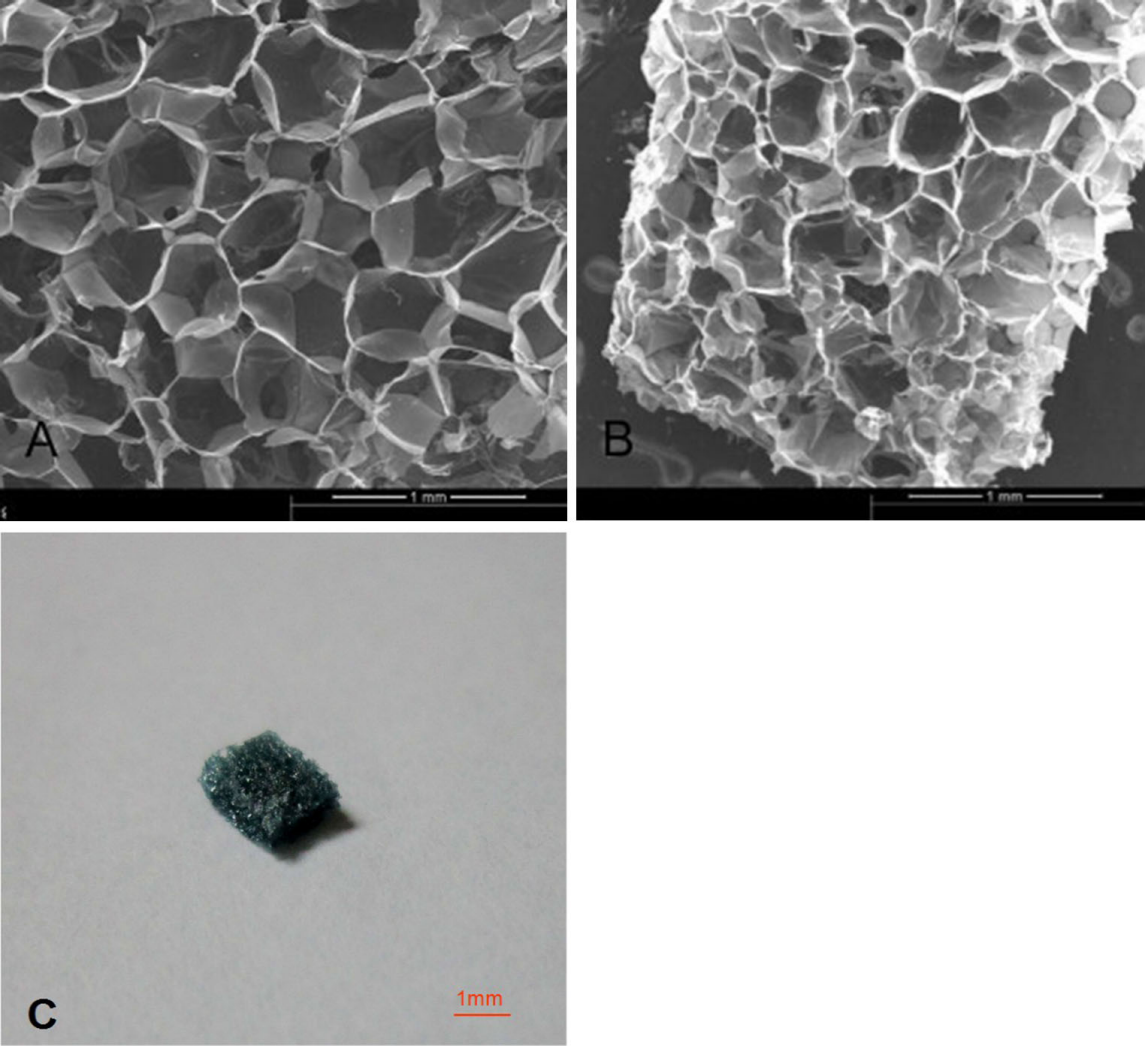
Scaffold characterisation. **a**, **b** SEM photomicrographs of the chitosan–gelatin scaffold. **c** Macroscopic aspects similar to a sponge

Through the measurement, calculating the porosity of chitosan–gelatin scaffold was above 90 %, and the degree of swelling was 25 %, the apparent density was 2.0 mg/mm^3^.The pore size of scaffold was between 100 and 400 μm. The macroscopic aspect and the three-dimensional architecture of the scaffold were similar to a sponge. Similar to trabecular bone, it consisted of a homogeneous porous-like arrangement (Fig. 3c). The SEM analysis revealed interconnected pores of different sizes and flat, relatively smooth walls (Fig. 3a, b).

### Characterization of the cell-scaffold construct

**Fig. 4:**
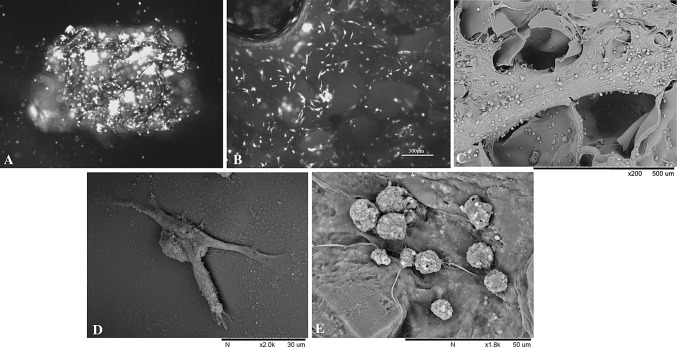
Characterization of the cell-scaffold construct. **a** The chitosan-gelatin scaffold seeded with GFP postive BMSCs. Before transplantation, the cell-scaffold construct was incubated for 48 h at 37 °C with 5 % CO_2_. **b** FDA- Pl staining of the cell-scaffold construct. **c**–**e** Scanning electron microscopy of the cell-scaffold construct

After 1 weeks’ culture, the scaffold was stained by FDA-Pl. Compared to a number of viable cells emitting green light, the loss of activity of the cells emitting red light are relatively few (Fig. 4a, b). A lot of dyed green fluorescent cells were clinging on the aperture, and showed a long typical fusiform as the morphology of stem cell peculiar. The PI was staining the nuclei of death cell, they were relatively few from Fig. 4, suggesting that morphology of BMSCs were normal after co-culturing, growing in good condition. BMSCs fused into cell clusters, covering internal and surface of the entire scaffold, so it could be that the chitosan–gelatin scaffold has non-toxic side effects on BMSCs.

SEM showed lots of cells attached to the scaffold widely, tight junction between cells and forming neurospheres similar to neural stem cells (Fig. 4c). Some cells were dividing, some cells secreted extracellular matrix, some cells extended many pseudopodia (Fig. 4b, c), so it could be that BMSCs were growing in a good condition.

### The repair of the defective spinal cord after cell-scaffold transplantation

**Fig. 5:**
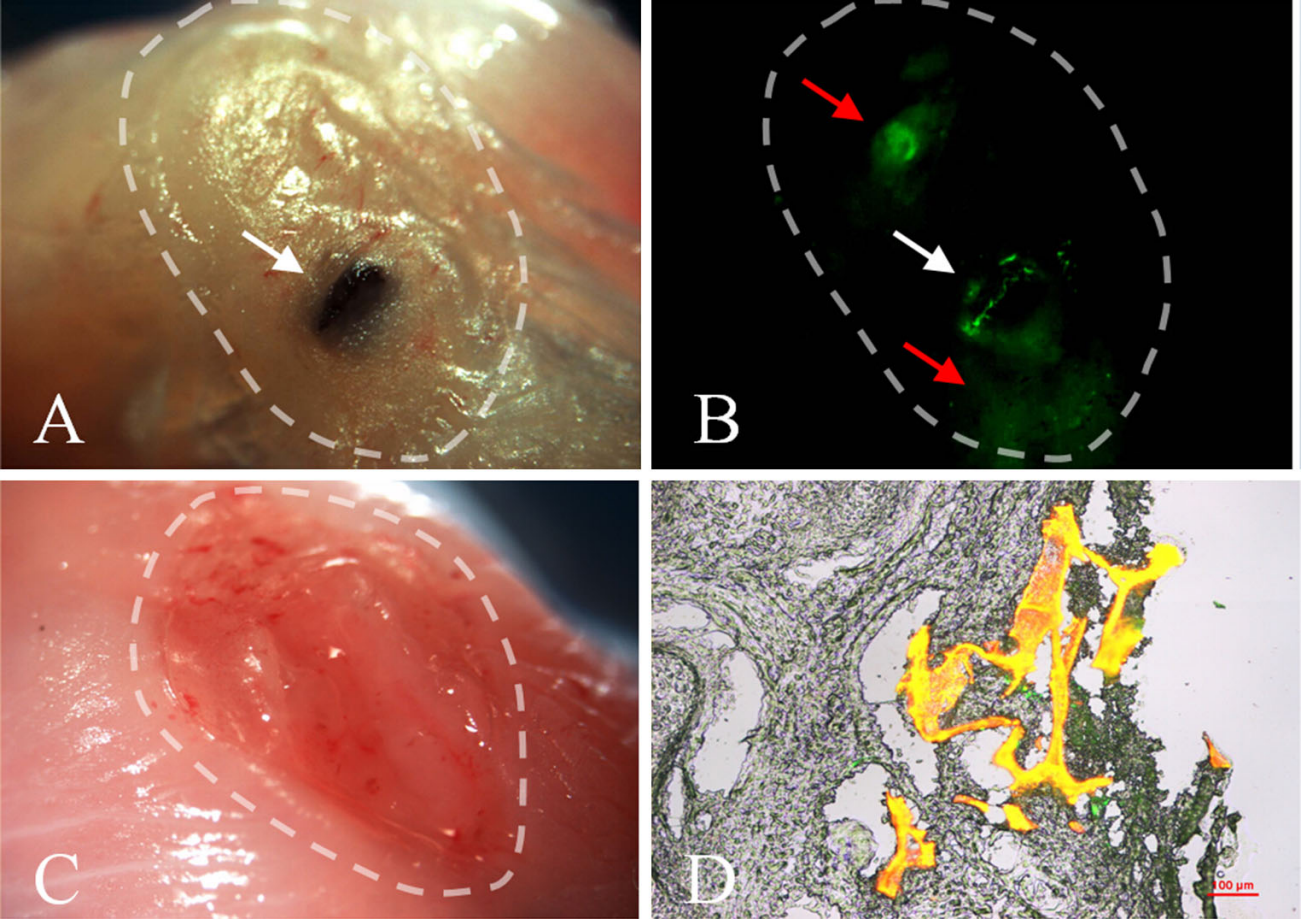
The repair of the defective spinal cord after cell-scaffold transplantation. **a** Stereomicroscopic imaging of fetus with spina bifida aperta showed the skin defect almost closed after cell-scaffold transplantation. The *black* object indicated by *arrow* was cell-scaffold construct. **b** Fluorescent stereomicroscopic imaging (same vision with figure **a**) showed the most of GFP labelled BMSCs were stayed in the scaffold (*white arrow*), and many GFP labelled BMSCs migrate outside to the scaffold and spread to the defective region of spina bifida aperta (*red arrow*). **c** Stereomicroscopic imaging of fetus with spina bifida aperta without cell-scaffold transplantation showed obvious defective spinal cord(view multiple was ×2). **d** Frozen transverse section of defective spinal cord with cell-scaffold (same fetus with Fig. 6a, the thickness of 20 μm), the *red* was the scaffold, the *green* was GFP labelled BMSCs (the scale was 100 μm)

**Fig. 6:**
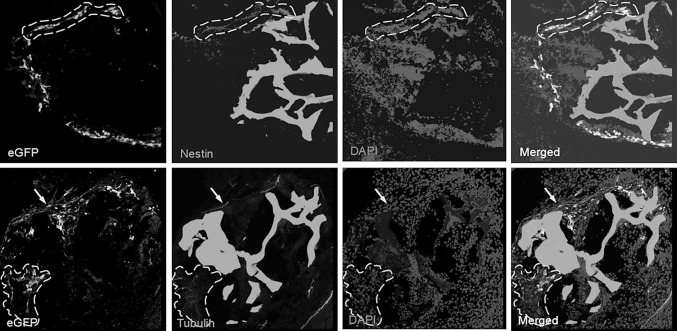
The differentiation of BMSCs in transplanted cell-scaffold construct in spinal column. The linear region is double positive cells, namely the GFP-BMSCs after transplantation in vivo differentiated into neural stem cells (Nestin) and neurons (Tubulin). (the vision are all in ×20)

The experiment was operated through intrauterine transplantation of cell-scaffold constructs on congenital spina bifida aperta fetal rats at E16. A total of 52 pregnant rats underwent surgery, one of them died in E20, another 51 pregnant rats which underwent surgery though cesarean section to obtain the fetal rats. A total of 134 fetal rats received cell-scaffold construct transplantation, of which 69 survived (survival rate was 51.5 %). The results found that the defective region of spinal cord in rat fetuses with spina bifida aperta at E20 decreased obviously after transplantation of the scaffold combined with BMSCs, and the skin defect almost closed compared with the fetus without transplantation under stereomicroscopy (Fig. 5a, c). Under fluorescent stereomicroscope most of GFP labelled BMSCs were stayed in the scaffold, and many GFP labelled BMSCs migrate outside of the scaffold and spread to the entire defective region of spina bifida aperta (Figs. 5b, 6). The results of frozen transverse sections showed that the defective tissue were filled with not only the transplanted chitosan–gelatin scaffold and GFP labeled BMSCs, but also many kinds of surrounding niche cells (Fig. 5d).

### Differentiation of BMSCs in transplanted cell-scaffold construct in spinal column of spina bifida aperta

To determine whether BMSCs in the scaffold differentiated into different cell types after transplantation in the defective region of fetal rats with spinal bifida aperta, the expression of specific cell markers were analyzed. During the 4 days′ time of embryonic development, transplanted BMSCs expressed neural stem cell specific surface markers (Nestin) and early neuron specific surface markers (Tubulin). These results indicated that the transplanted BMSCs in the rat fetuses could differentiate into neural stem cell and neurons, suggesting that prenatal transplantation of the scaffold combined with MSCs could have the potential to treat spinal cord defect in fetuses with spina bifida aperta by the regeneration of neurons.

## Discussion

Biological material is an important field of tissue engineering, it serves as a carrier of seed cells, providing a three-dimensional space for nutrition metabolism, proliferation and differentiation and the secretion of extracellular matrix of seed cells. As tissue engineering material, there must be lots of characteristics, besides stable quality, non-toxic and no adverse reaction, there are also compatibility and proper pore size and porosity [33]. The pore size of scaffold affects the organization reconstruction, reports in the literature have indicated aperture diameter of 20 μm in favor of the growth of some fibroblast cells, aperture of 20–125 μm in favor of the skin regeneration in adult mammals [11], the pore size of chitosan–gelatin scaffolds was 300 μm, can fully meet the demand of the tissue regeneration. High porosity of scaffold are conducive to nutrients′ entering and discharge of metabolic waste, the uniform pore size, particular size of the scaffold is suitable for the growth of different cells. The pore size has a direct influence on the cell, pore size and connectivity is the key for the design of material structure and cell adhesion tissue [29, 35]. In addition, chemical composition of chitosan–gelatin scaffolds consisted uniquely of carbon and oxygen, which indicates that degradation product of scaffold is harmless to the organization [36]. FDA as a fluorochrome, can make active cell cytoplasm staining, the experiment choose FDA to evaluate the activity of cells in the scaffold, while PI can make the nuclear of cell death staining, the results found that the vision under the fluorescence microscope is full of green fluorescent spindle cells, and only visible minority death cells dying red. The cells with normal morphology in attached to the scaffold can also be found under electron microscope, around the cells with a large amount of extracellular matrix, and mitotic cells can be observed. This series showed that BMSCs in the scaffold grew in good condition, the chitosan–gelatin scaffold had good biocompatibility.

Congenital malformations affect approximately 5 % of all live births every year [44, 45]. Several of these are associated with in utero foetal demise, significant neonatal morbidity and mortality. Despite early diagnosis of the condition in most of cases, treatment options for the affected fetuses are still limiting and not satisfactory. Currently, MMC repair operation in utero is being offered to selected mothers in several centres, and over 330 such interventions have been performed since 2000 [46–51]. It was reported that prenatal repair of the spinal cord defect greatly reduced the mortality of the birth defect, suggesting that attenuation of the progress of the damage of defective spinal cord at the early stage of pregnancy is beneficial to the fetus survival. However, if the defect involves too large an area, MMC repair operation cannot achieve an ideal result. The use of a tissue engineering scaffold seeded with stem cells can largely overcome this issue. In the present study, we first combined application of tissue engineering scaffolds and microsurgery technology in utero transplantation, we transplanted cell-scaffold construct to the spinal cord of congenital spina bifida aperta fetal rat to study whether this method can play a role in defect tissue regeneration of the spinal bifida fetal rat. The results found that the defective region of spinal cord in rat fetuses with spina bifida aperta at E20 decreased obviously, and the skin defect almost closed compared with the fetus without transplantation. Under fluorescent stereomicroscope we found that most of GFP labelled BMSCs were stayed in the scaffold, and many of them migrate outside of the scaffold and spread to the entire defective region of spina bifida aperta. This characteristic would be valuable for the repair of serious defects, especially for the spina bifida aperta which have the defects of not only spinal cord but also skin, muscle and vertebra. Chitosan–gelatin scaffold characterization revealed the porous structure, which indicates that these matrices are highly interconnected to allow nutrient exchange and waste removal [52]. The scaffold has some biological characteristics, such as bleeding, pain, wound healing, and has the permeability to oxygen, can reduce scar formation, help wound healing [53–55]. In a certain degree, the large range of spinal cord repair of spina bifida fetal rats are related to the properties of the scaffold. Mesenchymal stem cells have the potential to differentiate into different cell types, being induced by the surrounding microenvironment to differentiate into many kinds of cell type after being transplanted in vivo. Our study used immunofluorescence in frozen transverse sections of spinal vertebra found that transplanted BMSCs in chitosan–gelatin scaffolds in vivo can be respectively differentiation into neuronal stem cells and neurons, and the defective tissue were filled with not only the transplanted chitosan–gelatin scaffold and GFP labelled BMSCs, but also many kinds of surrounding niche cells. Mesenchymal stem cells can differentiate into neuronal cells on a tissue engineered nerve scaffold [56]. Our previous studys’ results show that MSCs survived, migrated and was able to differentiate into neural lineage cells [18, 19]. This study further confirms the characteristics of bone marrow mesenchymal stem cells. Neural damages in fetus with spina bifida aperta were occurred at early stage of development, so it is necessary to use stem cell replacement therapy as early as possible. In the present study, we performed intrauterine transplantation of cell-scaffold constructs at E16, in the future study it would performed at earlier stage of development (E14 or E15). It can make stem cells have more time to migration and differentiation after transplantation, and the spinal cord defect of spina bifida can be better reconstructed. In future studies, the long-term study about the function of rat fetuses with spina bifida aperta should be performed.

With the development of stem cell technology and tissue engineering, major components of regenerative medicine have made significant progress in many fields, providing a new approach and tremendous potential for spina bifida aperta repair and reconstruction. Although the application of mesenchymal stem cells further expands the source of seed cells, the legal and ethical issues remain unsolved. Therefore, the applications of tissue engineering and stem cell technology are beginning to enter clinical practice, and some clinical trials should see a wide prospect. However, the studies should take time to provide realistic follow-up data before the method can be applied to human disorders. We maintain the view that stem cell technology and tissue engineering -based strategies for the treatment of spina bifida aperta are in the early stages and the move from basic experiments to clinical applications will remain a long and difficult conversion.
